# Phase II Clinical Trial of GM-CSF Treatment in Patients with Hormone-Refractory or Hormone-Naïve Adenocarcinoma of the Prostate

**DOI:** 10.4137/cmo.s566

**Published:** 2008-07-08

**Authors:** Robert J. Amato, Joan Hernandez-McClain

**Affiliations:** Genitourinary Oncology Program, The Methodist Hospital Research Institute, Houston, TX

**Keywords:** prostate-specific antigen, prostate cancer, androgen independent, hormone therapy, immunotherapy, GM-CSF

## Abstract

The objective of this Phase II clinical trial was to determine the effects of chronic GM-CSF dosing on PSA levels in men with hormone-refractory or hormone-naïve prostate cancer. Six hormone-refractory and 10 hormone-naïve patients were recruited from an institutional practice and were treated with 250 and 125 μg/m^2^ of GM-CSF, respectively, 3 times per week for continuous 12-week treatment cycles until evidence of disease progression, as indicated by 2 consecutive rising PSA levels. PSA levels were measured every 6 weeks. Of the 6 hormone-refractory patients, 2 were classified with progressive disease after 4 months and 1 after 1.75 months. The best PSA responses for the remaining 3 patients were 3%, 12%, and 32% declines which lasted from 1.75 to 8.5 months. Of the 10 hormone-naïve patients, 2 were classified with progressive disease after 3 and 12 months, and 1 patient met the criteria for stable disease after 7.75 months. The best PSA response for the remaining 7 patients ranged from 7% to 42% declines which lasted from 0.5 to 10 months. These results indicate that further study of GM-CSF administration is not warranted for hormone-refractory patients but is recommended for hormone-naïve patients using a chronic dosing regimen.

## Introduction

Currently, no standard treatment exists for advanced prostate cancer patients with biochemical relapse (BCR) as defined by rising PSA levels after definitive local therapy. Three-year follow-up data extracted from the caPSURE database revealed that BCR occurred in 27% of patients after radiation therapy, 22% of patients after cryotherapy, and 14% of the patients after radical prostatectomy.^1^ Furthermore, BCR occurred in approximately one third of prostate cancer patients within 10 years of radical prostatectomy.^2^,^3^ While hormone therapy has provided clinical benefit for 24–36 months when initiated at the time of clinical evidence of metastatis,^2^ it remains palliative with extension of 5-year survival rates by merely 2%–3% in select high-risk patients^4^ and remains the last treatment option after local therapy failure.^2^,^5^ In addition, patients may be reluctant to begin hormone therapy treatment due to many adverse side effects, including decreased muscle mass, loss of libido, hot flashes, mild anemia, and increased risk of osteoporosis,^6^ and some patients who receive early hormone therapy develop androgen-independent rising PSA levels while still asymptomatic and without radiographic evidence of disease progression.^2^ Other therapeutic treatments for rising PSA include observation alone or radiation treatment to the prostate or prostate bed. Since few effective treatments are available for patients with PSA-only progression and because persistent post-treatment PSA levels are indicative of poor prognosis, these patients make ideal candidates for clinical trials of novel drug treatments designed to reduce PSA levels.

Several clinical trials have shown that immune modulation by cytokines may be a promising therapy for malignancies.^7^ The cytokine GM-CSF regulates granulocyte and macrophage differentiation and function and has been shown to induce dendritic cell antitumor activity.^8^ Several recent clinical trials have reported that treating hormone-refractory patients with GM-CSF alone or in combination with thalidomide is mildly effective at decreasing PSA levels.

The current study reports the results of a Phase II clinical trial which examined the effects of GM-CSF on PSA levels in patients with BCR after failure of primary treatment with radiotherapy, radical prostatectomy, or both. The PSA responses to GM-CSF treatment of both hormone-refractory patients (progressive disease despite hormone therapy) and hormone-naïve patients (no prior hormone therapy) were measured to determine whether use of the GM-CSF regimen warrants further study in both groups.

## Patients and Methods

This study was approved by the Institutional Review Board Committee at Baylor College of Medicine. Patient eligibility criteria included histological confirmation of adenocarcinoma of the prostate, disease progression evidenced by 2 consecutive rises in PSA levels over 4 weeks (with or without radiographic involvement), life expectancy of at least 3 months, and Zubrod performance status >2. For the hormone refractory patients, additional eligibility criteria included failure of conventional hormonal treatment, antiantigen withdrawal, and serum testosterone levels of ≤50 ng/dl. Medically castrated patients continued testicular suppression therapy during enrollment. For hormone naïve patients, testosterone levels of >200 ng/dl were required. The patients did not receive concurrent chemotherapy or immunotherapy. No hormone refractory patient received more than 1 cytotoxic therapy from which they were fully recovered (at least 6 weeks) prior to enrollment. Each patient had an absolute peripheral granulocyte count ≥1500, a platelet count ≥100 000, bilirubin ≤1.5 mg/dl, serum glutamic pyruvic transaminase ≤2 times the upper limits of normal, and a serum creatinine ≤1.5 mg/dl. All patients gave written informed consent prior to initiation of study treatment.

Prior to treatment, a complete medical history, physical examination, chest x-ray, bone scan, and CT scan of the abdomen and pelvis were performed. In addition, each patient underwent a complete blood count with differential, platelet count, urinalysis, albumin, alkaline phosphatase, AST calcium, LDH, total bilirubin, BUN, creatinine, inorganic phosphorus, electrolytes, PSA, CEA, testosterone, PT, and PTT measurements. For each 12-week treatment cycle, hormone-refractory patients were subcutaneously injected with 250 μg/m^2^ GM-CSF and hormone-naïve patients were injected with 125 μg/m^2^ 3 times per week with at least 24 hours between injections. Serum PSA levels were measured every 6 weeks. When an elevated PSA was found, a confirmatory measurement was taken 2 weeks later. At the end of each 12-week treatment cycle, serum chemistry profiles, bone scans, plain films, CT scans, and physical exams were performed. At least 3 treatment cycles were completed unless intolerable toxicity (grade 3 or 4) developed, as evaluated using the NCI Common Toxicity Criteria, Version 2.0.^9^ Twelve-week treatment cycles were continued until disease progression.

Responses to treatment were classified as (1) complete response if PSA normalized and radiographic evidence of disease resolution was maintained for at least 8 weeks; (2) partial response if a >50% decrease in the sum of the products of all measurable lesions and a >50% PSA decline without normalization was maintained for 12 weeks; (3) stable disease if the criteria for complete response, partial response, or progressive disease were not met (i.e. no disease progression or improvement); and (4) progressive disease if 3 consecutive increases in PSA to >25% above the nadir were measured at least 2 weeks apart, if the sum of the products of any measurable lesions or the estimated size of nonmeasurable lesions increased by >25%, or if new lesions appeared.

## Statistical Methods

The primary objectives were to assess PSA response to treatment with GM-CSF in a group of hormone refractory patients and a separate group of hormone naïve patients. A decline in PSA level of ≥50% from baseline was considered to be of special clinical interest. Secondary objectives included assessment of overall survival and treatment duration. Overall survival was calculated from the date of enrollment to the date of death. Treatment duration was calculated as the time between the first treatment and the time the patient was taken off the study (i.e. stopped treatment). Duration of PSA response was defined as the time from the first PSA decline of >50% to PSA > 25% above the nadir.

The study design called for recruiting a maximum of 22 patients in both groups. Eight responses among hormone-refractory patients or 15 responses in the hormone-naïve patients would warrant further study of GM-CSF in these populations, respectively. However, this trial was closed before the targeted number of patients was enrolled because none of the hormone-refractory patient achieved sufficient PSA declines to warrant continuing the study. Based on the encouraging responses we observed in hormone-naïve patients, along with promising results achieved in studies of combination therapy of GM-CSF and thalidomide in this population,^2^ hormone-naïve patients were moved into a trial of GM-CSF plus thalidomide.

## Results

Ten patients with hormone-naïve adenocarcinoma of the prostate were enrolled and treated. Their median baseline PSA was 6.20 ng/ml and ranged from 2.6 to 14.2 ng/ml (Table 1). One patient had both bone and nodal metastases. Members of this group participated in the study for a median of 7.9 months (Table 3). Median overall survival was 47.6 months and ranged from 41.5 to 52 months. Two patients developed progressive disease after 1 and 4 treatment cycles, respectively. One patient met the criteria for stable disease designation after 7.75 months. For the 7 remaining patients, the best PSA response ranged from 7% to 42% decline.

Six patients were enrolled and treated in the hormone refractory arm. Median baseline PSA was 32 ng/ml and ranged from 2.6–42.6 ng/ml (Table 2). One patient had bone metastasis. The median duration of enrollment was 5.8 months (1.75 to 18.25 months) for hormone-refractory patients (Table 3). Median overall survival was 38.1 months and ranged from 19.75 to 51.5 months. Two patients met the criteria for progressive disease designation after 4 months of enrollment and one at 1.75 months. For the remaining 3 patients, the best PSA response ranged from 3% to 32% decline.

Nonhematologic toxicity as a result of GM-CSF administration was minimal (Table 4); grade 1 edema, fever, chills, bruising, herpes zoster, and pain were each reported by one patient. One patient had grade 3 thrombosis. Common hematologic toxicities included grade 1 hyperglycemia, grade 2 leukopenia, and grade 2 elevated leukocytes. One patient each had grade 3 leukopenia, grade 3 neutropenia, and grade 4 neutropenia (Table 4).

## Discussion

Rising PSA levels after primary treatment for prostate cancer are problematic as definitive treatment regimens have not yet been established. There is no compelling evidence that hormone treatment improves survival for patients with rising PSA levels^4^, and the PSA level at which hormone treatment should begin is not known. Adverse effects significantly affect quality of life and may make patients reluctant to begin or continue hormone therapy. Furthermore, a subgroup of patients has been shown to develop androgen-independent prostate cancer after hormone treatment. Thus, treatment decisions must balance the need for disease management with the patient’s quality of life and overall survival.

Recently, use of GM-CSF has been examined as a means to halt rising PSA levels. In a Phase II clinical trial study, Dreicer et al. reported only 1 hormone-refractory patient out of 9 with a PSA response that was ≥50% and 5 patients with PSA responses that were <50% when treated with 250 μg/m^2^ GM-CSF 3 times per week for 6 months. In addition, 3 patients were classified with progressive disease after 4 months of treatment.^10^ When treated daily with 250 μg/m^2^ GM-CSF for 14 days followed by 250 μg/m^2^ GM-CSF 3 times per week until disease progression, only 1 of 13 hormone-refractory patients had a 6-week sustained PSA decline of ≥50%, while 12 patients experienced a PSA decline with a median of 32% (no range was reported).^11^ Interestingly, 5 of 22 hormone-refractory patients had ≥50% PSA decline when the 250 μg/m^2^ GM-CSF 3 times per week regimen was combined with a maximum daily dose of 200 μg of thalidomide,^2^ suggesting that the combination of an immunostimulatory agent with anti-inflammatory agent may be of more benefit to hormone-refractory patients.

In the current study, 3 of the 6 patients recruited for the hormone-refractory arm had progressive disease designations within 4 months of systemically administered GM-CSF treatment. The best PSA response of the remaining 3 patients was a 32% decline that lasted for 8.5 months of treatment. Results among the few hormone-refractory patients treated in this study do not appear as promising as the earlier reports. For this reason, we closed this trial early and do not recommend further study of the effects of GM-CSF on PSA levels in hormone-refractory patients at this time.

Schwaab et al. examined the immune response to GM-CSF administration and found little GM-CSF effect on PSA-specific CD+ and CD8+ T cell precursors. Seven of 14 patients experienced ≥25% drops in PSA levels and 1 had a ≥50% decline, but prior hormone treatment status of these patients was not reported. They concluded that administration of GM-CSF alone produced little therapeutic benefit and was unable to induce a PSA-specific T cell immunity; however, the existent PSA immunity in these patients suggests that they may benefit from immune-stimulatory therapies.^12^

In the current study, treatment of hormone-naïve patients with 125 μg/m^2^ GM-CSF 3 times per week produced a better response. While 2 patients had progressive disease designations and 1 was classified with stable disease, 7 patients had <50% PSA decline that lasted from 0.5–10 months. Dreicer et al. had similar results when hormone-naïve patients were treated with 250 μg/m^2^ GM-CSF 3 times per week for up to 6 months. One patient had disease progression while 5 had <50% PSA decline.^10^

These data from our study and others suggest that GM-CSF has some biological activity in patients with rising PSA levels. The promising results of GM-CSF/thalidomide combination treatment of hormone-refractory patients^2^ combined with the response of PSA-only, hormone-naïve patients to treatment with GM-CSF in the current study suggest that thalidomide may augment the effects of GM-CSF in PSA-only, hormone-naïve patients. This hypothesis is currently being investigated.

## Conclusion

This report provides further evidence of the efficacy of GM-CSF in preventing rising PSA levels in hormone naïve patients with PSA-only advanced prostate cancer. These results indicate that further study of GM-CSF administration to hormone-refractory patients is not warranted, but further study of a chronic dosing regimen for GM-CSF administration to hormone-naïve patients is recommended.

## Figures and Tables

**Table 1:** Patient characteristics within the hormone-naïve arm.

	Median (range)	% (n)
Age, years	64.0 (53–83)	
Primary therapy		
Radical prostatectomy		60 (6)
Radical prostatectomy + radiotherapy		10 (1)
Radiotherapy + gene therapy		10 (1)
Radiotherapy		20 (2)
Baseline PSA	6.20 (2.6–14.2) ng/ml	
Metastatic Disease		
PSA only		90 (9)
Bone		0 (0)
Bone and Nodal		10 (1)

**Table 2:** Patient characteristics within the hormone-refractory arm.

	Median (range)	% (n)
Age, years	76 (59–87)	
Primary therapy		
Radical prostatectomy		33 (2)
Radical prostatectomy + radiotherapy		33 (2)
Radiotherapy		33 (2)
Baseline PSA	32 (2.6–42.6) ng/ml	
Metastatic Disease		
PSA only		83 (5)
Bone		16 (1)
Bone and Nodal		0 (0)

**Table 3:** Patient response characteristics.

Arm	Time on study median (range) months	Best PSA response median% (range)	PSA duration median (range) months	Overall survival median (range) months
Hormone naïve	7.9 (3–14)	10 (7–42)*	2.66 (0–10)	47.55 (41.5–52)
Hormone refractory	5.8 (1.75–18.25)	12 (3–32)†	1.9 (0–8.5)	38.1 (19.75–51.5)

* n = 7, 2 patients had progressive disease and 1 patient was classified as stable.

† n = 3, 3 patients had progressive disease.

**Table 4:** Toxicities.

	Grade 1	Grade 2	Grade 3	Grade 4
Non-Hematologic Toxicities				
Edema	1	0	0	
Fever	1	0	0	
Chills	1	0	0	
Bruising	1	0	0	
Pain	1	0	0	
Thrombosis	0	0	1	
Hematologic toxicities				
Leukopenia	2	5	1	0
Neutropenia	0	3	1	1
Lymphopenia	0	1	0	0
Hyperglycemia	6	1	0	0
Neutrophilia	3	2	0	0
Anemia	2	0	0	0
Elevated Leukocytes	1	5	0	0
Eosinophilia	1	1	0	0

## References

[1] Grossfeld GD, Stier DM, Flanders SC (1998). Use of second treatment following definitive local therapy for prostate cancer: data from the caPSURE database. J Urol.

[2] Dreicer R, Klein EA, Elson P (2005). Phase II trial of GM-CSF + thalidomide in patients with androgen-independent metastatic prostate cancer. Urol Oncol.

[3] Pound CR, Partin AW, Eisenberger MA (1999). Natural history of progression after PSA elevation following radical prostatectomy. JAMA.

[4] Prostate Cancer Trialists’ Collaborative Group (2000). Maximum androgen blockade in advanced prostate cancer: an overview of the randomised trials. Lancet.

[5] Boccon-Gibod L, Djavan WB, Hammerer P (2004). Management of prostate-specific antigen relapse in prostate cancer: a European Consensus. Int J Clin Pract.

[6] Ward JF, Moul JW (2005). Rising prostate-specific antigen after primary prostate cancer therapy. Nat Clin Pract Urol.

[7] Waller EK, Ernstoff MS (2003). Modulation of antitumor immune responses by hematopoietic cytokines. Cancer.

[8] Rini BI, Weinberg V, Bok R, Small EJ (2003). Prostate-specific antigen kinetics as a measure of the biologic effect of granulocyte-macrophage colony-stimulating factor in patients with serologic progression of prostate cancer. J Clin Oncol.

[9] Dreicer R, See WA, Klein EA (2001). Phase II trial of GM-CSF in advanced prostate cancer. Invest New Drugs.

[10] National Cancer Institute (1999). Common Terminology Criteria for Adverse Events, version 2.0.

[11] Small EJ, Reese DM, Um B (1999). Therapy of advanced prostate cancer with granulocyte macrophage colony-stimulating factor. Clin Cancer Res.

[12] Schwaab T, Tretter CP, Gibson JJ (2006). Tumor-related immunity in prostate cancer patients treated with human recombinant granulocyte monocyte-colony stimulating factor (GM-CSF). Prostate.

